# Supplementary material for: Cancer Related Subarachnoid Hemorrhage: A Multicenter Retrospective Study Using Propensity Score Matching Analysis
Source: Front Cell Neurosci. 2022 Feb 7;16:813084. doi: 10.3389/fncel.2022.813084 (PMC8859848; doi:10.3389/fncel.2022.813084)
Supplement: Supplementary file 1 [file Data_Sheet_1.docx]

**Appendix: Table S1│**Data of subarachnoid hemorrhage onset in patients with cancer ^a^

| Characteristic | All patients,  n=82 | Solid tumor, n=21 | Liquid tumor,  n=40 | Brain tumor,  n=21 |
| --- | --- | --- | --- | --- |
| Hunt & Hess grade |  |  |  |  |
| Grade 1 | 33(40.20) | 9(42.90) | 15 (37.50) | 9 (42.90) |
| Grade 2 | 16(19.50) | 1(4.80) | 8 (20) | 7 (33.30) |
| Grade 3 | 14(17.10) | 2(9.50) | 9 (22.50) | 3 (14.30) |
| Grade 4 | 6(7.30) | 5(23.80) | 0 (0.00) | 1 (4.80） |
| Grade 5 | 13(15.90) | 4(19.40) | 8 (20) | 1 (4.80） |
| Time interval between cancer diagnosis and SAH onset |  |  |  |  |
| SAH as the first presentation of cancer | 13(15.90) | 5(23.80) | 5(12.50) | 3(14.30) |
| SAH onset after cancer |  |  |  |  |
| 1-180 days | 47(57.30) | 10 (47.60） | 25 (62.50) | 12 (57.10) |
| 181-360 days | 10(12.20) | 2(9.50) | 4 (10) | 4(19.40) |
| > 360 days | 12(14.60) | 4(19.40) | 6 (15) | 2(9.50) |

^a^ Data are expressed as no. (%). Percentages are rounded to the nearest decimal point and thus may not add up to 100. Abbreviations: SAH: subarachnoid hemorrhage.

**Appendix: Table S2│**Univariate analysis of continuous variable relating to 30-day survival rate in Original 82 cancer patients with SAH

| Variable | β | p Value | HR | 95.0% CI | |
| --- | --- | --- | --- | --- | --- |
|  |  |  |  | Lower quartile | Upper quartile |
| WBC | 0.007 | ＜0.01 | 1.007 | 1.003 | 1.011 |
| RBC | -0.181 | 0.149 | 0.834 | 0.652 | 1.067 |
| HGB | -0.009 | 0.039 | 0.991 | 0.982 | 1.000 |
| PLT | -0.002 | 0.195 | 0.998 | 0.995 | 1.001 |
| NC | 0.006 | 0.007 | 1.006 | 1.002 | 1.011 |
| NP | 0.034 | 0.950 | 1.034 | 0.359 | 2.982 |
| LY | 0.019 | 0.193 | 1.019 | 0.991 | 1.048 |
| LYP | -0.106 | 0.885 | 0.900 | 0.214 | 3.784 |
| TBIL | 0.020 | 0.049 | 1.021 | 1.000 | 1.042 |
| ALT | 0.011 | 0.007 | 1.011 | 1.003 | 1.019 |
| AST | 0.010 | ＜0.01 | 1.010 | 1.005 | 1.014 |
| GGT | 0.002 | 0.046 | 1.002 | 1.000 | 1.005 |
| TP | -0.019 | 0.311 | 0.981 | 0.946 | 1.018 |
| Alb | -0.037 | 0.015 | 0.964 | 0.936 | 0.993 |
| GLB | -0.003 | 0.611 | 0.997 | 0.984 | 1.009 |
| PAB | -0.007 | ＜0.01 | 0.993 | 0.989 | 0.997 |
| CCR | -0.007 | 0.123 | 0.993 | 0.985 | 1.002 |
| Cr | 0.002 | 0.006 | 1.002 | 1.001 | 1.004 |
| BUN | 0.030 | 0.005 | 1.031 | 1.009 | 1.053 |
| UA | 0.002 | 0.012 | 1.002 | 1.000 | 1.003 |
| PT | 0.085 | ＜0.01 | 1.089 | 1.042 | 1.138 |
| INR | 0.156 | 0.023 | 1.169 | 1.022 | 1.337 |
| APTT | 0.019 | 0.024 | 1.019 | 1.003 | 1.036 |
| TT | 0.013 | 0.142 | 1.013 | 0.996 | 1.030 |
| FIB | 0.014 | 0.865 | 1.014 | 0.860 | 1.196 |

Abbreviations: B=regression coefficient; OR=odds ratio; 95% CI=95% confidence interval; SAH=subarachnoid hemorrhage; WBC= white blood cell; RBC=red blood cell; HGB= hemoglobin; PLT= platelet; NC=neutrophil count; NP= neutrophil percentage; LY=lymphocyte; LYP=lymphocyte percentage; TBIL=total bilirubin; ALT=alanine transaminase; AST=aspartate transaminase; GGT=gamma-glutamyl transferase; TP= total protein; Alb= serum albumin; GLB=Globulin; PAB=prealbumin; CCR= creatinine clearance rate; Cr=creatinine; BUN= blood urea nitrogen; UA= uric acid; PT= prothrombin time; INR= international normalized ratio; APTT= activated partial thromboplastin time; TT= thrombin time; FIB=fibrinogen.

**Appendix: Table S3│**Univariate analysis of categorical variable relating to 30-day survival rate in Original 82 cancer patients with SAH

| Variable | | Log Rank χ^2^ | p |
| --- | --- | --- | --- |
| Gender | Female | 0.61 | 0.43 |
|  | Male |  |  |
| Solid tumor | No | 1.24 | 0.26 |
|  | Yes |  |  |
| Liquid tumor | No | 3.04 | 0.08 |
|  | Yes |  |  |
| Brain tumor | No | 6.35 | 0.01 |
|  | Yes |  |  |
| Previous Stroke | No | 0.02 | 0.89 |
|  | Yes |  |  |
| Hypertension | No | 0.10 | 0.76 |
|  | Yes |  |  |
| Diabetes | No | 7.20 | 0.01 |
|  | Yes |  |  |
| Atrial fibrillation | No | 4.22 | 0.04 |
|  | Yes |  |  |
| Coronary heart disease | No | 0.05 | 0.82 |
|  | Yes |  |  |
| Smoking | No | 1.41 | 0.24 |
|  | Yes |  |  |
| Drinking | No | 0.01 | 0.92 |
|  | Yes |  |  |
| Hemiparesis | No | 2.35 | 0.13 |
|  | Yes |  |  |
| Headache | No | 0.21 | 0.64 |
|  | Yes |  |  |
| Disturbance of consciousness | No | 11.68 | 0.01 |
|  | Yes |  |  |
| Encephalopathy | No | 5.52 | 0.02 |
|  | Yes |  |  |
| Vomit | No | 8.23 | 0.01 |
|  | Yes |  |  |
| Seizure | No | 0.35 | 0.56 |
|  | Yes |  |  |
| Aphasia | No | 5.66 | 0.02 |
|  | Yes |  |  |
| Hemisensory disturbance | No | 2.22 | 0.14 |
|  | Yes |  |  |
| Hydrocephalus | No | 0.03 | 0.87 |
|  | Yes |  |  |
| Cerebral hernia | No | 7.85 | ＜0.01 |
|  | Yes |  |  |
| Coma | No | 11.64 | ＜0.01 |
|  | Yes |  |  |
| ITH | No | 1.20 | 0.27 |
|  | Yes |  |  |
| Coagulopathy | No | 4.29 | 0.03 |
|  | Yes |  |  |
| ITH and coagulopathy | No | 0.03 | 0.87 |
|  | Yes |  |  |
| Other causes of SAH | No | 4.19 | 0.04 |
|  | Yes |  |  |
| Reduce intracranial pressure | No | 2.50 | 0.11 |
|  | Yes |  |  |
| Anti-infection | No | ＜0.01 | 0.97 |
|  | Yes |  |  |
| Radiotherapy | No | 0.22 | 0.64 |
|  | Yes |  |  |
| Chemotherapy | No | 2.92 | 0.09 |
|  | Yes |  |  |
| Surgical treatment | No | 6.53 | 0.01 |
|  | Yes |  |  |
| Hormone therapy | No | 0.22 | 0.64 |
|  | Yes |  |  |

Abbreviations: SAH=subarachnoid hemorrhage.

| Variable | B | p Value | HR | 95% CI | |
| --- | --- | --- | --- | --- | --- |
|  |  |  |  | Lower quartile | Upper quartile |
| WBC | 0.007 | ＜0.01 | 1.007 | 1.003 | 1.011 |
| RBC | -0.138 | 0.212 | 0.871 | 0.701 | 1.082 |
| HGB | -0.007 | 0.060 | 0.993 | 0.985 | 1.000 |
| PLT | -0.002 | 0.207 | 0.998 | 0.996 | 1.001 |
| NC | 0.007 | 0.004 | 1.007 | 1.002 | 1.011 |
| NP | 0.701 | 0.253 | 2.015 | 0.607 | 6.694 |
| LY | 0.013 | 0.337 | 1.013 | 0.986 | 1.042 |
| LYP | -0.588 | 0.409 | 0.555 | 0.137 | 2.245 |
| TBIL | 0.018 | 0.059 | 1.019 | 0.999 | 1.038 |
| ALT | 0.010 | 0.018 | 1.010 | 1.002 | 1.018 |
| AST | 0.009 | ＜0.01 | 1.009 | 1.005 | 1.014 |
| GGT | 0.009 | ＜0.01 | 1.009 | 1.005 | 1.014 |
| TP | -0.018 | 0.295 | 0.982 | 0.950 | 1.016 |
| Alb | -0.023 | 0.108 | 0.977 | 0.949 | 1.005 |
| GLB | -0.005 | 0.431 | 0.995 | 0.981 | 1.008 |
| PAB | -0.005 | 0.001 | 0.995 | 0.991 | 0.998 |
| CCR | -0.005 | 0.233 | 0.995 | 0.988 | 1.003 |
| Cr | 0.002 | 0.006 | 1.002 | 1.001 | 1.004 |
| BUN | 0.029 | 0.006 | 1.030 | 1.009 | 1.052 |
| UA | 0.002 | 0.019 | 1.002 | 1.000 | 1.003 |
| PT | 0.085 | ＜0.01 | 1.089 | 1.042 | 1.137 |
| INR | 0.156 | 0.023 | 1.169 | 1.022 | 1.337 |
| APTT | 0.017 | 0.044 | 1.018 | 1.001 | 1.035 |
| TT | 0.011 | 0.236 | 1.011 | 0.993 | 1.029 |
| FIB | 0.037 | 0.627 | 1.038 | 0.894 | 1.205 |

**Appendix: Table S4│** Univariate analysis of continuous variable relating to 90-day survival rate in Original 82 cancer patients with SAH

Abbreviations: B=regression coefficient; OR=odds ratio; 95% CI=95% confidence interval; SAH=subarachnoid

hemorrhage; WBC= white blood cell; RBC=red blood cell; HGB= hemoglobin; PLT= platelet; NC=neutrophil count; NP= neutrophil percentage; LY=lymphocyte; LYP=lymphocyte percentage; TBIL=total bilirubin; ALT=alanine transaminase; AST=aspartate transaminase; GGT=gamma-glutamyl transferase; TP= total protein; Alb= serum albumin; GLB=Globulin; PAB=prealbumin; CCR= creatinine clearance rate; Cr=creatinine; BUN= blood urea nitrogen; UA= uric acid; PT= prothrombin time; INR= international normalized ratio; APTT= activated partial thromboplastin time; TT= thrombin time; FIB=fibrinogen.

**Appendix: Table S5│**Univariate analysis of categorical variable relating to 90-day survival rate in Original 82 cancer patients with SAH

| Variable | | Log Rank χ^2^ | p Value |
| --- | --- | --- | --- |
| Gender | Female | 0.63 | 0.43 |
|  | Male |  |  |
| Solid tumor | NO | 0.30 | 0.58 |
|  | YES |  |  |
| Liquid tumor | NO | 4.46 | 0.03 |
|  | YES |  |  |
| Brain tumor | NO | 4.65 | 0.03 |
|  | YES |  |  |
| Previous Stroke | NO | 0.04 | 0.84 |
|  | YES |  |  |
| Hypertension | NO | 0.33 | 0.56 |
|  | YES |  |  |
| Diabetes | NO | 7.20 | 0.01 |
|  | YES |  |  |
| Atrial fibrillation | NO | 4.22 | 0.04 |
|  | YES |  |  |
| Coronary heart disease | NO | 0.02 | 0.89 |
|  | YES |  |  |
| Smoking | NO | 0.20 | 0.65 |
|  | YES |  |  |
| Drinking | NO | 0.03 | 0.87 |
|  | YES |  |  |
| Hemiparesis | NO | 2.17 | 0.14 |
|  | YES |  |  |
| Headache | NO | 0.52 | 0.47 |
|  | YES |  |  |
| Disturbance of consciousness | NO | 13.64 | ＜0.01 |
|  | YES |  |  |
| Encephalopathy | NO | 7.83 | 0.01 |
|  | YES |  |  |
| Vomit | NO | 9.78 | ＜0.01 |
|  | YES |  |  |
| Seizure | NO | 0.01 | 0.91 |
|  | YES |  |  |
| Aphasia | NO | 6.26 | 0.01 |
|  | YES |  |  |
| Hemisensory disturbance | NO | 1.31 | 0.25 |
|  | YES |  |  |
| Hydrocephalus | NO | 0.03 | 0.87 |
|  | YES |  |  |
| Cerebral hernia | NO | 7.57 | ＜0.01 |
|  | YES |  |  |
| Coma | NO | 11.29 | ＜0.01 |
|  | YES |  |  |
| ITH | NO | 1.47 | 0.22 |
|  | YES |  |  |
| Coagulopathy | NO | 3.88 | 0.04 |
|  | YES |  |  |
| ITH and Coagulopathy | NO | 0.14 | 0.70 |
|  | YES |  |  |
| Other cause of SAH | NO | 2.81 | 0.09 |
|  | YES |  |  |
| Plasma or platelet transfusion | NO | 0.05 | 0.82 |
|  | YES |  |  |
| Reduce intracranial pressure | NO | 3.23 | 0.07 |
|  | YES |  |  |
| Anti-infection | NO | ＜0.01 | 0.97 |
|  | YES |  |  |
| Radiotherapy | NO | 0.19 | 0.66 |
|  | YES |  |  |
| Chemotherapy | NO | 3.54 | 0.06 |
|  | YES |  |  |
| Surgical treatment | NO | 6.84 | ＜0.01 |
|  | YES |  |  |
| Hormone therapy | NO | 0.22 | 0.64 |
|  | YES |  |  |

Abbreviations: SAH=subarachnoid hemorrhage.

Appendix: Table S6**│**Predictors of 30- and 90-day mortality via Cox regression models

| Variable | 30-Day mortality | | | 90-Day mortality | | |
| --- | --- | --- | --- | --- | --- | --- |
|  | HR | 95% CI | p Value | HR | 95% CI | p Value |
| Brain tumor | 0.26 | 0.05-1.32 | 0.10 | NS | NS | NS |
| Having liquid tumor | NS | NS | NS | 4.13 | 1.17-6.5 | 0.03 |
| Diabetes | 2.20 | 0.28-17.25 | 0.45 | 2.13 | 0.33-13.87 | 0.43 |
| Atrial fibrillation | 4.57 | 1.48-6.04 | 0.03 | 11.13 | 0.62-20.70 | 0.10 |
| Disturbance of consciousness | 1.62 | 0.44-5.92 | 0.46 | 2.15 | 0.78-5.92 | 0.13 |
| Encephalopathy | 0.46 | 0.05-4.34 | 0.50 | 0.98 | 0.15-6.22 | 0.98 |
| Vomit | 2.64 | 0.90-7.69 | 0.08 | 2.34 | 0.98-5.57 | 0.05 |
| Having aphasia | 2.30 | 1.08-4.96 | 0.04 | 4.75 | 0.91-24.73 | 0.06 |
| Cerebral hernia | 1.64 | 0.42-6.44 | 0.48 | 1.10 | 0.35-3.46 | 0.86 |
| Coma | 0.80 | 0.18-3.59 | 0.77 | 0.78 | 0.25-2.38 | 0.66 |
| Having coagulopathy | 1.72 | 1.5-3.77 | 0.03 | 1.81 | 1.02-3.85 | 0.01 |
| Cryptogenic SAH | 2.10 | 1.52-3.54 | 0.04 | 2.11 | 1.05-3.16 | 0.03 |
| Surgical treatment | 1.68 | 0.35-8.03 | 0.51 | 1.32 | 0.38-4.58 | 0 .65 |
| WBC | 0.99 | 0.57-1.02 | 0 .59 | 0.99 | 0.97-1.01 | 0.36 |
| HGB | 0.99 | 0.57-1.01 | 0.43 | NS | NS | NS |
| NC | 1.01 | 0.69-1.02 | 0.60 | 1.00 | 0.99-1.02 | 0.35 |
| TBIL | 0.97 | 0.93-1.01 | 0.13 | NS | NS | NS |
| ALT | 1.01 | 0.10-1.03 | 0 .15 | 1.01 | 0.99-1.02 | 0.06 |
| AST | 1.01 | 0.10-1.01 | 0.19 | 1.00 | 0.99-1.01 | 0.28 |
| GGT | 1.61 | 1.51-1.83 | 0.01 | 1.00 | 1.00-1.00 | 0.07 |
| Alb | 1.02 | 0.97-1.08 | 0.48 | NS | NS | NS |
| PAB | 0.68 | 0.45-0.90 | ＜0.01 | 0.69 | 0.58-0.92 | ＜0.01 |
| Cr | 1.00 | 0.99-1.00 | 0.92 | 1.00 | 0.99-1.01 | 0.62 |
| BUN | 1.03 | 0.99-1.08 | 0.17 | 1.02 | 0.98-1.06 | 0.17 |
| UA | 1.00 | 0.99-1.00 | 0.86 | 1.00 | 0.99-1.01 | 0.70 |
| PT | 0.97 | 0.86-1.08 | 0.54 | 0.97 | 0.88-1.07 | 0.60 |
| INR | 1.11 | 0.82-1.49 | 0.49 | 1.14 | 0.86-1.51 | 0.34 |
| APTT | 1.82 | 1.42-3.10 | 0.03 | 1.65 | 1.51-3.09 | 0.01 |

Abbreviations: HR= hazard ratio; 95% CI=95% confidence interval; SAH=subarachnoid hemorrhage; NS =not significant; WBC= white blood cell; HGB= hemoglobin; NC=neutrophil count; TBIL=total bilirubin; ALT=alanine transaminase; AST=aspartate transaminase; GGT=gamma-glutamyl transferase; TP= total protein; Alb= serum albumin; PAB=prealbumin; Cr=creatinine; BUN= blood urea nitrogen; UA= uric acid; PT= prothrombin time; INR= international normalized ratio; APTT= activated partial thromboplastin time.

**Appendix: Table S7│**Multivariate logistic regression analysis of variables relating to the onset of SAH in Original 391 Patients with cancer

| Variable | β | p Value | OR | 95% CI |
| --- | --- | --- | --- | --- |
| Male | 0.347 | 0.355 | 1.415 | 0.678-2.951 |
| Having previous stroke | 1.924 | 0.006 | 3.848 | 1.733-5.056 |
| Hypertension | 0.757 | 0.051 | 2.132 | 0.997-4.560 |
| Current smoker | -0.204 | 0.683 | 0.815 | 0.306-2.175 |
| Current drinking | -0.520 | 0.723 | 0.595 | 0.034-10.534 |
| Brain tumor | 0.255 | 0.545 | 1.291 | 0.564-2.953 |
| WBC | -0.003 | 0.291 | 0.997 | 0.991-1.003 |
| RBC | -0.417 | 0.226 | 0.659 | 0.336-1.295 |
| HGB | 0.011 | 0.373 | 1.011 | 0.987-1.036 |
| PLT | -0.003 | 0.849 | 1.000 | 0.997-1.003 |
| NP | 0.817 | 0.342 | 2.263 | 0.419-12.219 |
| LY | -0.005 | 0.708 | 0.995 | 0.968-1.022 |
| LYP | -0.031 | 0.855 | 0.969 | 0.693-1.356 |
| TBIL | -0.013 | 0.423 | 0.987 | 0.955 -1.019 |
| GGT | 0.001 | 0.928 | 1.000 | 0.997-1.004 |
| PAB | -0.001 | 0.591 | 0.999 | 0.994-1.004 |
| UA | 0.003 | 0.060 | 0.997 | 0.995-1.001 |
| PT | 0.133 | 0.028 | 1.142 | 1.015-1.286 |
| INR | 0.895 | 0.396 | 2.447 | 0.310-19.333 |
| Constant | -3.844 | 0.011 | 0.021 |  |

Abbreviations: SAH=subarachnoid hemorrhage; WBC= white blood cell; RBC=red blood cell; HGB= hemoglobin; PLT= platelet; NP= neutrophil percentage; LY=lymphocyte; LYP=lymphocyte percentage; TBIL=total bilirubin; GGT=gamma-glutamyl transferase; PAB=prealbumin; UA= uric acid; PT= prothrombin time; INR= international normalized ratio; B=regression coefficient; OR=odds ratio; 95% CI=95% confidence interval.

**Appendix****: Table S8│** Multivariate analysis of variables relating to the onset of SAH in 150 patients with cancer after propensity score matching

| Variable | β | p Value | OR | 95% CI |
| --- | --- | --- | --- | --- |
| PLT | -0.010 | ＜0.001 | 0.869 | 0.692-0.981 |
| LY | -0.020 | 0.307 | 0.979 | 0.939-1.020 |
| LYP | -1.474 | 0.141 | 0.229 | 0.032-1.633 |
| ALT | -0.013 | 0.267 | 0.987 | 0.965-1.010 |
| AST | -0.011 | 0.173 | 0.989 | 0.973-1.005 |
| UA | -0.016 | 0.051 | 0.994 | 0.991-1.108 |
| PT | 0.444 | ＜0.001 | 1.559 | 1.229 -1.976 |
| INR | 1.384 | 0.277 | 3.992 | 0.329-48.405 |
| Constant | 1.866 | 0.033 | 6.462 |  |

Abbreviations: SAH=subarachnoid hemorrhage; PLT=platelet; LY=lymphocyte; LYP=lymphocyte percentage; ALT=alanine transaminase; AST=aspartate transaminase; UA= uric acid; PT= prothrombin time; INR= international normalized ratio; β=regression coefficient; OR=odds ratio; 95% CI=95% confidence interval.

**Appendix: Table S9│**Area under ROC curves for predicting the onset of SAH.

| Variable | PT | PLT | JPF |
| --- | --- | --- | --- |
| AUC | 0.78 | 0.77 | 0.81 |
| SE | 0.04 | 0.04 | 0.04 |
| p Value | <0.01 | <0.01 | <0.01 |
| 95% CI | 0.71-0.86 | 0.69-0.85 | 0.75-0.88 |
| Sensitivity (%) | 66.70 | 62.70 | 65.30 |
| Specificity (%) | 78.70 | 92.00 | 89.30 |
| Youden index | 0.45 | 0.55 | 0.55 |
| Cut-off value | 13.45 | 89.40 | 11.72 |

Abbreviations: PLT=platelet; PT= prothrombin time; JBF= joint predictive factor; AUC =Area under the curve; SE= standard error; CI=confidence interval.
